# A Bibliometric Analysis of the Trends and Impact of Neuromarketing Research: Peering Into the Consumer Brain

**DOI:** 10.7759/cureus.69314

**Published:** 2024-09-13

**Authors:** Lekshmi S, Melby Joseph, Bobby Simon, Sibichan Joseph, Gibin Jacob, Alan Lukose

**Affiliations:** 1 Research and PG Department of Commerce, Government College Attingal, Attingal, IND; 2 Department of Business Administration, Marian College Kuttikkanam Autonomous, Kuttikkanam, IND; 3 Department of Commerce, St. Thomas College, Palai, Palai, IND; 4 Department of Commerce, St. Paul's College, Kalamassery, Kalamassery, IND; 5 Department of Commerce, Prajyoti Niketan College, Pudukad, Thrissur, IND

**Keywords:** bibliometric analyses, biblioshiny, brain-based marketing, citespace, neuromarketing

## Abstract

Neuromarketing is the application of neuroscience and cognitive science to understand and influence consumer behavior and the different underlying decision-making processes. This neuromarketing bibliometric study uses Scopus for bibliographic data and Biblioshiny and Citespace for the overall analysis. Annual scientific production is considered to outline some general lines of critical trends in volume over time. It provides an overview of the most relevant authors and their contributions and creates co-citation networks with the cited authors to identify influential researchers and collaborative networks. Sources that are most relevant to the discussion, together with co-citation patterns, show important articles and journals. Mapping countries' scientific production and collaboration show the manifold contributions of single countries to global research and how some topics can be created out of cooperation. Trend topics were analyzed to detect emerging research themes; factorial analysis helped in the actual clustering of the research topics. It identifies keywords with the most robust citation bursts, meaning times of the most concentrated research activity and emerging areas of interest. The research identifies gaps and produces some practical implications, putting forward a roadmap for future research directions and the necessity of advanced computational techniques in neuromarketing research.

## Introduction and background

Neuromarketing is a multi-disciplinary tool that combines neuroscience and marketing, providing in-depth and descriptive information about consumer behavior that is often overlooked with the use of traditional marketing techniques [1,2]. In other words, by exploring the sub-conscious processes that drive consumer decisions for product purchase, neuromarketing helps companies with research-based strategies for marketing, improved advertisement practices, and message creation [2]. Neuromarketing is the application of rules and techniques derived from neuroscience and cognitive psychology to marketing. It aims to reveal customer needs and motivations, their preferences, instances, and scenarios, which traditional methods of marketing, specifically surveys and focus groups, simply cannot catch [1,3]. Traditional marketing is very much reliant on self-reported data; it is biased and may not fully take into account the subconscious factors that drive consumer behavior. On the other hand, neuromarketing relies on the sensorimotor, cognitive, and affective responses in the brain to marketing stimuli, thereby revealing all aspects of consumer choice [4].

Neuromarketing employs various methods and approaches that measure consumer behavior. Among the most essential tools are brain-imaging methods, referred to as neuroimaging and physiological tracking [5]. Brain imaging -namely, functional magnetic resonance imaging (fMRI) and electroencephalography (EEG) - measures brain activity and provides real-time data on how consumers respond to marketing messages [5,6]. As such, the mentioned techniques give a deeper understanding of immediate unconscious reactions to marketing stimuli. Physiological tracking methods - like eye tracking, heart rate monitoring, and skin conductance - capture consumers' immediate and involuntary responses to marketing stimuli in more precise and comprehensive ways, focusing on how consumers perceive and react to marketing elements [7,8].

Neuromarketing offers several advantages over conventional marketing research: a deeper understanding of consumer behavior, improved marketing effectiveness, reduced uncertainty associated with the product, and optimization of product design [9]. In essence, neuromarketing makes possible research in the field of subconscious responses to allow a more comprehensive understanding of what drives customer decisions [10]. Now that everything is more transparent, businesses can see what resonates with consumers to come up with more powerful advertisements, product designs, and overall customer experience [3]. Insights from neuromarketing may lead to the design of products that respond better to the needs and preferences of the consumer.

Neuromarketing can provide beneficial information but raises several ethical considerations that need to be taken into account by companies and researchers: Questions on privacy and consent, potential manipulation, and choice of appropriate partners [11]. Data collected from neuromarketing studies often measures susceptible data from the subjects' brain activity and physiological responses. Special and apparent attention should be paid to the necessity of proper informed consent from the subjects and ensure the most careful and confidential data handling. The application of neuroscience to influence consumer behavior raises ethical questions about manipulativeness [3,12]. It calls for careful and responsible operationalization by business organizations in ways that make marketing processes clear and run in a manner that respects the autonomy of the consumer [12,13].

Neuromarketing has been widely used in advertising campaign optimization, package design, web design, shopping outlets, and political campaigns [14]. For instance, features of emotional engagement related to buying associated with Coca-Cola and Pepsi fMRI and EEG have both been used, enabling them to develop ads that can captivate and be more effective [15]. This research has also been conducted in e-commerce companies to help optimize their web design and user experience [16]. Neuromarketing has been applied to physical retail environments where the protocol will monitor the consumers' physiological response to store layouts, lighting conditions, and music [17]. It has extended to its application even in political campaigns, gauging the voters' emotional reactions to various speeches, ads, and debates of multiple candidates [18].

The field of neuromarketing is continuously evolving, with new technologies and methodologies emerging [19]. Future directions include advancements in brain imaging, integration with big data, personalized marketing, and the development of ethical frameworks [19]. As brain imaging technology advances, researchers will obtain more detailed and accurate data on neural responses to marketing stimuli [19]. The integration of neuromarketing data with big data analytics holds significant potential [20]. Neuromarketing can contribute to personalized marketing strategies by understanding individual consumers' neural and emotional responses [21]. The development of ethical frameworks and guidelines will be essential to ensuring that neuromarketing practices are conducted responsibly and transparently [22].

There has been significant growth in both research and the application of neuromarketing, which is indicative of growing fascination with the increasing literature dedicated to exploring ways neuroscientific tools and techniques can help enhance understanding and influence consumer behavior. Bibliometric analysis is one way of systematically mapping this evolving research landscape [23]. This paper explores academic literature through a quantitative approach, and its analysis in neuromarketing reveals the research trends, who the key authors in the field are, what the key publications are, and what emerging issues relate to the topic of study [24]. In a broader context, bibliometric analysis identifies how general knowledge or our understanding of neuromarketing, in this case, is being developed or disseminated, who the key contributors to such development are, and how change in research topics has evolved.

Biblioshiny and Citespace are powerful tools for conducting bibliometric analysis. Biblioshiny is freely available, open-source software designed for user-friendly web-based bibliometric analysis [25]. It enables the user to import bibliographic data, perform various analyses, and finally generate intuitive graphical representations [26]. Biblioshiny allows researchers to carry out citation analysis, co-citation analysis, and keyword co-occurrence analysis toward the final identification of influential papers, links among works, and commonly occurring themes within the literature [27]. Citespace is a Java-based application that provides a visual and analytical tool for detecting patterns and trends in the scientific literature [28]. The tool looks to identify critical points of development in some fields, including pivotal publications, research fronts, and intellectual turning points [29]. Beyond these functions, Citespace also serves advanced visualization methodologies that make apparent underlying patterns and trends by generating visual maps of the networks of co-citations and performing burst detection to determine topics of rising interest.

The objectives of the bibliometric analysis on neuromarketing are multifaceted. The analysis aims to map the chronological development of neuromarketing research, highlighting key trends and shifts in focus areas over time. It also seeks to pinpoint the most cited authors and journals, thereby identifying the pivotal contributions that have shaped the field. Additionally, it aims to examine the collaborative relationships among researchers and countries, revealing the primary networks and hubs of neuromarketing research. The analysis will also detect emerging topics and research fronts within neuromarketing by analyzing trend topics, factorial analysis, and keyword citation bursts, indicating new directions in the field. Finally, it will identify underexplored areas and research gaps in the existing literature, providing a basis for future studies to address these gaps and advance the field.

## Review

Materials and methods

The bibliographic database used for this bibliometric analysis on neuromarketing was Scopus, chosen for its extensive collection of scientific literature [30]. The keyword "neuromarketing" was utilized in the search, yielding 1,115 results without any language restrictions. The inclusion criteria for this analysis were documents categorized as journal articles, conference papers, and book chapters, as these formats typically contain original research, substantial reviews, or comprehensive studies. The data were then refined by applying exclusion criteria, which removed reviews, editorials, letters, notes, and short surveys from the dataset. Following this process, 983 documents were selected for further analysis. The selected documents were saved in a CSV file format. Bibliometric analysis was performed using two software tools: Biblioshiny and Citespace. Biblioshiny provided an easy-to-use web interface for conducting comprehensive analyses and visualizations. Citespace, Java-based software, was used to visualize trends and patterns in the scientific literature, identify critical developments, and map collaborative networks within the neuromarketing research community.

Findings and discussions 

Main Aspects of the Investigation

Table 1 displays the key information regarding the bibliometric analysis of neuromarketing. The investigation covers publications from 2005 to 2024, utilizing 567 sources, including journals, books, and other types of publications, and analyzing 983 documents. The field of neuromarketing shows an annual growth rate of 24.37%, with documents averaging 4.9 years of age and receiving an average of 14.65 citations each. The total number of references cited across all documents is 40,835. In terms of document content, 2,534 keywords were identified using Keywords Plus, and the authors provided 2,181 unique keywords. The analysis includes contributions from 2,483 authors, with 130 single-authored documents and an average of 3.41 co-authors per document, while 20.24% of the documents involved international collaborations. The document types analyzed comprise 616 articles, 137 book chapters, and 230 conference papers.

**Table 1:** Main information of the investigation

Description	Results
Main information about data	
Timespan	2005:2024
Sources (Journals, Books, etc.)	567
Documents	983
Annual growth rate %	24.37
Document average age (years)	4.9
Average citations per doc	14.65
References	40835
Document contents	
Keywords plus (ID)	2534
Author's keywords (DE)	2181
Authors	
Authors	2483
Authors of single-authored docs	130
Authors collaboration	
Single-authored docs	141
Co-authors per doc	3.41
International co-authorships %	20.24
Document types	
Article	616
Book chapter	137
Conference paper	230

Annual Scientific Productions

**Figure 1:**
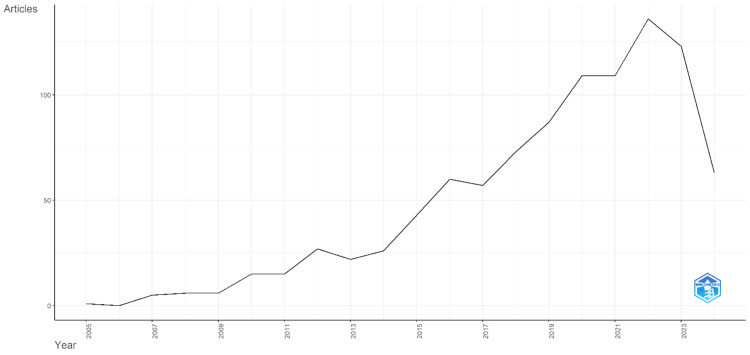
Annual scientific productions

The annual scientific production in Figure 1 shows a significant increase over the years from 2005 to 2024. In 2005, only one article was published, and no publications were recorded in 2006. However, from 2007 onward, the number of articles steadily rose, reaching five in 2007 and six in 2008 and 2009. There was a noticeable jump in 2010 and 2011, with 15 articles each year. This upward trend continued, with 27 articles in 2012 and 22 in 2013; 2014 to 2016 saw a more pronounced increase, with articles numbering 26, 43, and 60, respectively. This growth trend continued, reaching 57 articles in 2017 and 73 in 2018. The following years saw further growth, with 87 articles in 2019 and a peak of 109 articles in 2020 and 2021. The most prolific year so far was 2022, with 136 articles. In 2023, the production slightly decreased to 123 articles. As of June 2024, 63 articles have been published, indicating that the year might see a comparable output to previous years if the trend continues. These data highlight the expanding interest and research activity in neuromarketing over the past two decades.

Most Relevant Authors

Table 2 shows the prolific authors based on the number of their papers in neuromarketing research. Babiloni F would score the most significant number of articles of publication, which is 29, illustrating how great science could be with such a contribution. The second one is Cherubino P. with 19; the third is Vecchiato G. with 15, clearly indicating the contribution's significance. It is true: Trettel A has 13 and Ma Q has 12 articles published; they are adept in neuromarketing research. Maglione AG and Singh J have ten articles, Alsharif AH has 11 papers, Salleh NZM has 9, and Gill R has eight articles published. These authors are important representatives of the neuromarketing literature, and their broad work probably models and likely influences current recognition and tendencies in the field.

**Table 2:** Most relevant authors

Authors	Articles
Babiloni F	29
Cherubino P	19
Vecchiato G	15
Trettel A	13
Ma Q	12
Alsharif AH	11
Maglione AG	10
Singh J	10
Salleh Nzm	9
Gill R	8

Timezone Network Visualization of Co-citation of Cited Authors

**Figure 2:**
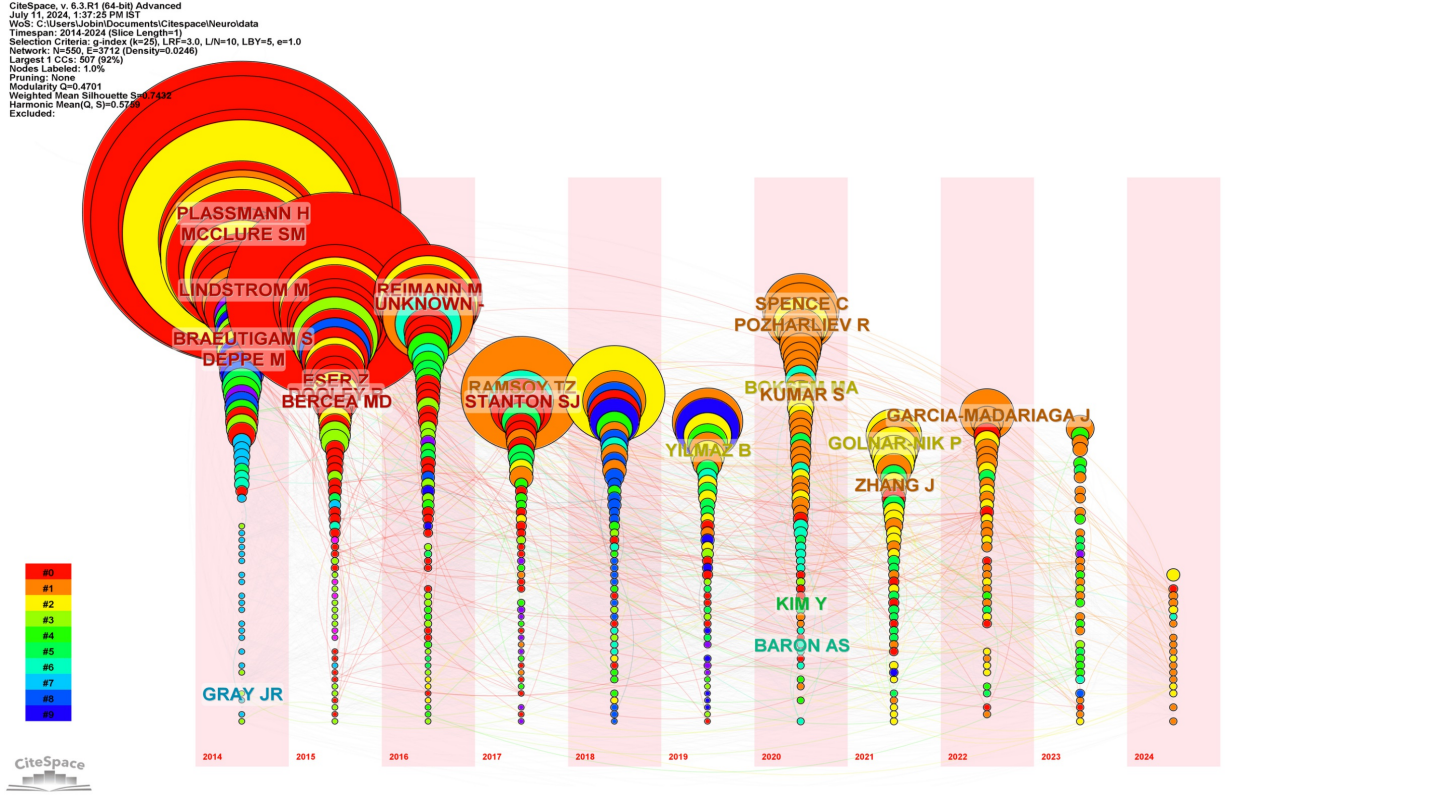
Timezone network visualization of co-citation of cited authors

Figure 2 illustrates the timezone network visualization of the co-citation of cited authors and reveals a comprehensive landscape divided into 12 distinct clusters, each with a unique thematic focus. Cluster #0, labeled "Strategic Planner," is the largest with 116 members and features prominent figures like Lee N (215 citations), Plassmann H (204 citations), and Ariely D (166 citations). Cluster #1, identified as "Comprehensive Bibliometric Analysis," follows with 94 members and includes key contributors such as Venkatraman V (109 citations), Ramsoy TZ (80 citations), and Smidts A (60 citations). Cluster #2, titled "EEG Signal," contains 62 members, with highly cited researchers like Vecchiato G (161 citations), Ohme R (109 citations), and Davidson RJ (77 citations). Cluster #3, "Typical Food Product," includes 45 members, highlighting Kotler P (57 citations), Damasio A (33 citations), and Bargh JA (19 citations). Cluster #4, "P300 Signal," has 44 members and features Bradley MM (35 citations), Bagozzi RP (26 citations), and Li S (25 citations). Cluster #5, "Consumers Attention," comprises 38 members, with leading figures such as Klimesch W and Wedel M, both with 25 citations, and Babiloni F (16 citations). In Cluster #6, "Strategic Tool," which has 27 members, the most cited authors include Berns GS (43 citations), Pieters R (42 citations), and Madan CR (20 citations). Cluster #7, labeled "O Abordare Exploratorie La," includes 24 members, with Farah MJ (11 citations), Huettel SA (9 citations), and Gazzaniga MS (9 citations) as key contributors. Cluster #8, "Gaining Information," has 23 members and features Zaltman G (47 citations), Fortunato VCR (35 citations), and Braidot N (25 citations). Cluster #9, "Future Research Agenda," includes 15 members, with top-cited members like Harris JM (42 citations), Bechara A (34 citations), and Daugherty T (32 citations). Cluster #10, "Case Studies," has 14 members, with influential researchers such as Damasio AR (36 citations), Balconi M (21 citations), and Mauss IB (8 citations). Finally, Cluster #11, "Consumer Behavior," is the smallest with five members, featuring Mlodinov L (3 citations), Ard A (2 citations), and Jung CG (2 citations). These clusters collectively map out the diverse and rich field of neuromarketing, illustrating the various research foci and the prominent scholars driving the discourse in each area.

Table 3 presents a co-citation analysis of cited authors, categorizing them into 12 distinct clusters based on their thematic focus.

**Table 3:** Co-citation of cited authors

ClusterID	No. of members	Silhouette	Label	Most cited members
0	116	0.672	Strategic planner	Lee N - 215, Plassmann H - 204, Ariely D - 166
1	94	0.627	Comprehensive bibliometric analysis	Venkatraman V - 109, Ramsoy TZ - 80, Smidts A - 60
2	62	0.805	EEG signal	Vecchiato G - 161, Ohme R - 109, Davidson RJ - 77
3	45	0.646	Typical food product	Kotler P - 57, Damasio A - 33, Bargh JA -19
4	44	0.729	p300 signal	Bradley MM - 35, Bagozzi RP - 26, LI S - 25
5	38	0.829	Consumers attention	Klimesch W - 25, Wedel M - 25, Babiloni F - 16
6	27	0.765	Strategic tool	Berns GS - 43, Pieters R - 42, Madan CR - 20
7	24	0.961	o abordare exploratorie la	Farah MJ - 11, Huettel SA - 9, Gazzaniga MS - 9
8	23	0.973	Gaining information	Zaltman G - 47, Fortunato VC - 35, Braidot N - 25
9	15	0.928	Future research agenda	Harris JM - 42, Bechara A - 34, Daugherty T - 32
10	14	0.873	Case studies	Damasio AR - 36, Balconi M - 21, Mauss IB - 8
11	5	0.999	Consumer behavior	Mlodinov L - 3, Ard A -2 J, Ung CG - 2

Most Relevant Sources

Table 4 lists the most impactful journals in the field of neuromarketing based on the number of publications. The journal Frontiers in Psychology leads with 26 articles, indicating its significant role in disseminating neuromarketing research. Following this, the Springer Proceedings in Business and Economics has 16 articles, showing its prominence in bridging business and neuromarketing topics. Frontiers in Human Neuroscience also plays a crucial role with 15 publications, highlighting its focus on the neuroscientific aspects of consumer behavior. Smart Innovation, Systems and Technologies, and Advances in Intelligent Systems and Computing both have 14 and 13 articles, respectively, showcasing their contributions to the technological and innovative approaches in neuromarketing. Developments in Marketing Science: Proceedings of the Academy of Marketing Science and Lecture Notes in Computer Science (including its subseries) each have 13 publications, indicating their importance in the academic and interdisciplinary discussions of neuromarketing. The Ethics and Neuromarketing: Implications for Market Research and Business Practice and Journal of Consumer Behavior both have 12 articles, reflecting their focus on the ethical considerations and consumer behavior aspects of neuromarketing. Lastly, Analyzing the Strategic Role of Neuromarketing and Consumer Neuroscience, which has 10 articles, emphasizes strategic applications and the integration of neuromarketing in broader consumer neuroscience research. These journals collectively represent the core sources where significant neuromarketing research is published, shaping the current understanding and future directions of the field.

**Table 4:** Most relevant sources

Sources	Articles
Frontiers in Psychology	26
Springer Proceedings in Business and Economics	16
Frontiers in Human Neuroscience	15
Smart Innovation, Systems and Technologies	14
Advances in Intelligent Systems and Computing	13
Developments in Marketing Science: Proceedings of The Academy of Marketing Science	13
Lecture Notes in Computer Science (Including Subseries Lecture Notes in Artificial Intelligence and Lecture Notes in Bioinformatics)	13
Ethics and Neuromarketing: Implications for Market Research and Business Practice	12
Journal of Consumer Behavior	12
Analyzing the Strategic Role of Neuromarketing and Consumer Neuroscience	10

Timezone Network Visualization of Co-citation of Cited Journals

**Figure 3:**
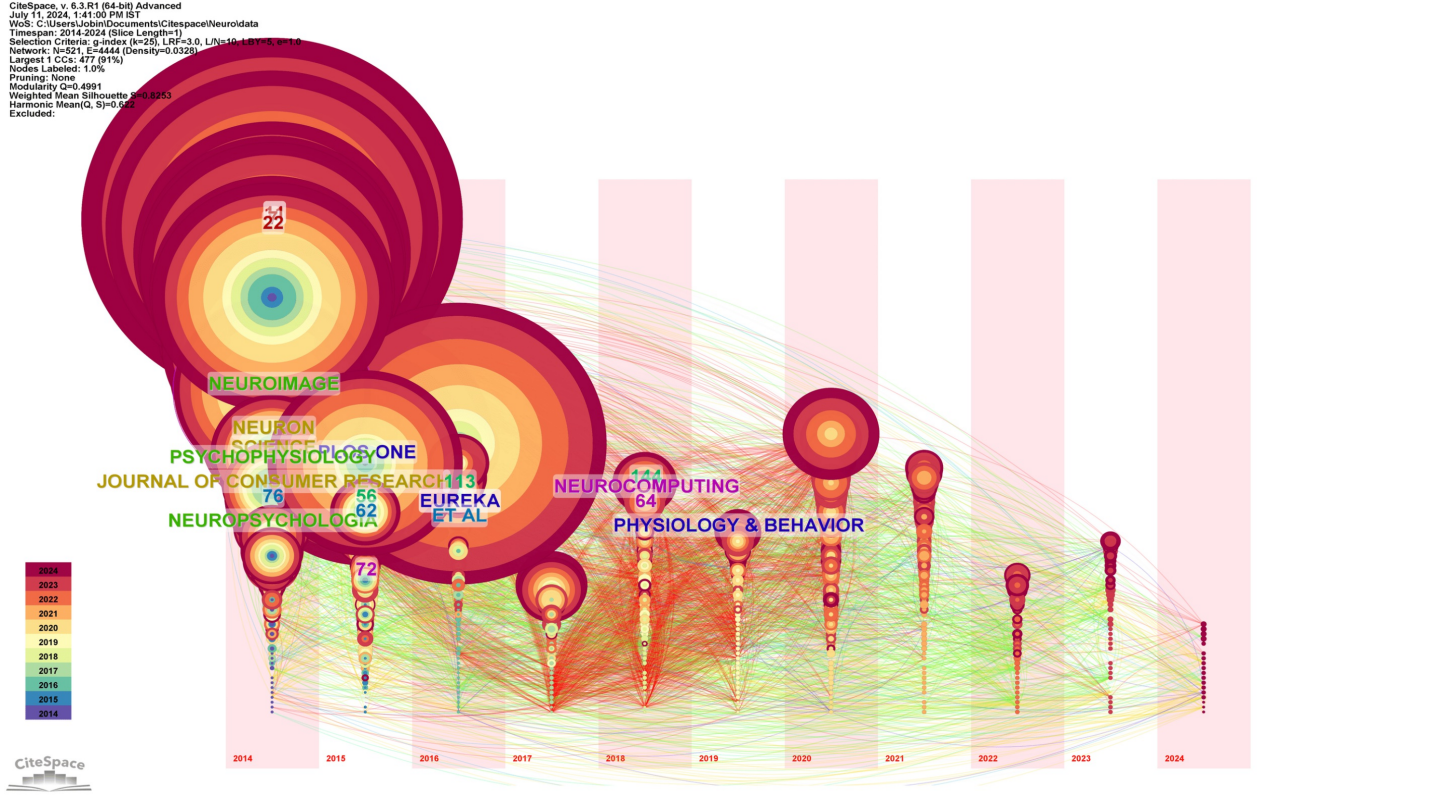
Timezone network visualization of co-citation of cited journals

Figure 3 depicts the timezone network visualization of co-citation among cited journals in neuromarketing research, which is organized into seven distinct clusters, each representing different thematic areas within the field. The largest cluster, Cluster #0: Advertising Effectiveness, comprises 112 members and is highly focused on the effectiveness of advertising through neuromarketing approaches. This cluster is characterized by a high silhouette value of 0.897, indicating well-defined boundaries. Although the most cited journals within this cluster are not provided, the major citing article is by Alsharif (2023), which discusses neuromarketing tools in the marketing mix. Cluster #1: Biomedical Technology is the second-largest cluster with 109 members and a silhouette value of 0.756. This cluster revolves around the intersection of neuromarketing and biomedical technology, particularly involving fMRI studies. The most cited journals in this cluster are Neuron (99 citations), Science (84 citations), and the Journal of Consumer Research (66 citations), highlighting the cross-disciplinary impact of this research area. The key citing article is by Rodríguez (2023), which reviews consumer neuroscience in branding and packaging. Cluster #2: Functional Near-Infrared Spectroscopy Study has 101 members and a silhouette value of 0.894, focusing on the application of functional near-infrared spectroscopy (fNIRS) and EEG signals in neuromarketing research. The top journals cited in this cluster include NeuroImage (134 citations), Psychophysiology (77 citations), and Neuropsychologia (53 citations), emphasizing the strong foundation in cognitive neuroscience. The primary citing article is by Wolf (2021), which explores the contribution of eye-tracking in studying cognitive impairments. The remaining clusters further diversify the thematic landscape of neuromarketing research. Cluster #3: Bibliometric Analysis, with 69 members, delves into meta-analytical approaches to understanding the field, while Cluster #4: Isolated Packaging Variable (46 members) explores the impact of packaging on consumer preferences. Cluster #5: Gaining information (22 members) is closely related to using psychophysiological approaches to gather consumer insights, with PLOS ONE (133 citations) being a key journal. Finally, Cluster #6: Unplanned Virtual Shopping (18 members) examines consumer behavior in virtual environments, with Neurocomputing (31 citations) as a notable publication. Each cluster highlights different facets of neuromarketing, ranging from foundational research in neuroscience to practical applications in consumer behavior and marketing strategies.

Table 5 summarizes the clusters identified in the timezone network visualization of co-citation among cited journals in neuromarketing research. This table helps us understand the thematic areas and the key journals contributing to each cluster.

**Table 5:** Co-citation of cited journals

ClusterID	No. of Members	Silhouette	Label	Most Cited Journals
0	112	0.897	Advertising effectiveness	International Journal of Consumer Studies
1	109	0.756	Biomedical technology	Neuron, Science, Journal of Consumer Research
2	101	0.894	Functional near-infrared spectroscopy study	Neuroimage, Psychophysiology, Neuropsychologia
3	69	0.72	Bibliometric analysis	International Journal of Information Management
4	46	0.768	Isolated packaging variable	Physiology and Behavior
5	22	0.929	Gaining information	PlosOne, Physiology and Behavior, Eureka
6	18	0.836	Unplanned virtual shopping	Neurocomputing

Countries' Scientific Production

Table 6 presents the number of scientific documents produced by different countries, indicating their contributions to the field. Spain leads with 332 documents, closely followed by Italy with 330, demonstrating significant research activity and interest in neuromarketing within these countries. The USA ranks third with 251 documents, highlighting its substantial contribution to the global neuromarketing research landscape. China (200 documents) and India (186 documents) also show strong involvement, reflecting growing interest and research output in neuromarketing in these rapidly developing economies. Malaysia (135 documents) and Turkey (129 documents) further contribute to the diversity of research contributions. Japan (112 documents), Germany (108 documents), and the UK (91 documents) round out the list, each with over 90 publications, underscoring their roles as active participants in advancing neuromarketing research. This distribution of scientific production highlights the global nature of neuromarketing research and the significant contributions from both Western and Asian countries.

**Table 6:** Countries' scientific production

Region	Number of documents
Spain	332
Italy	330
Usa	251
China	200
India	186
Malaysia	135
Turkey	129
Japan	112
Germany	108
Uk	91

Timeline Network Visualization of Countries’ Collaborations

**Figure 4:**
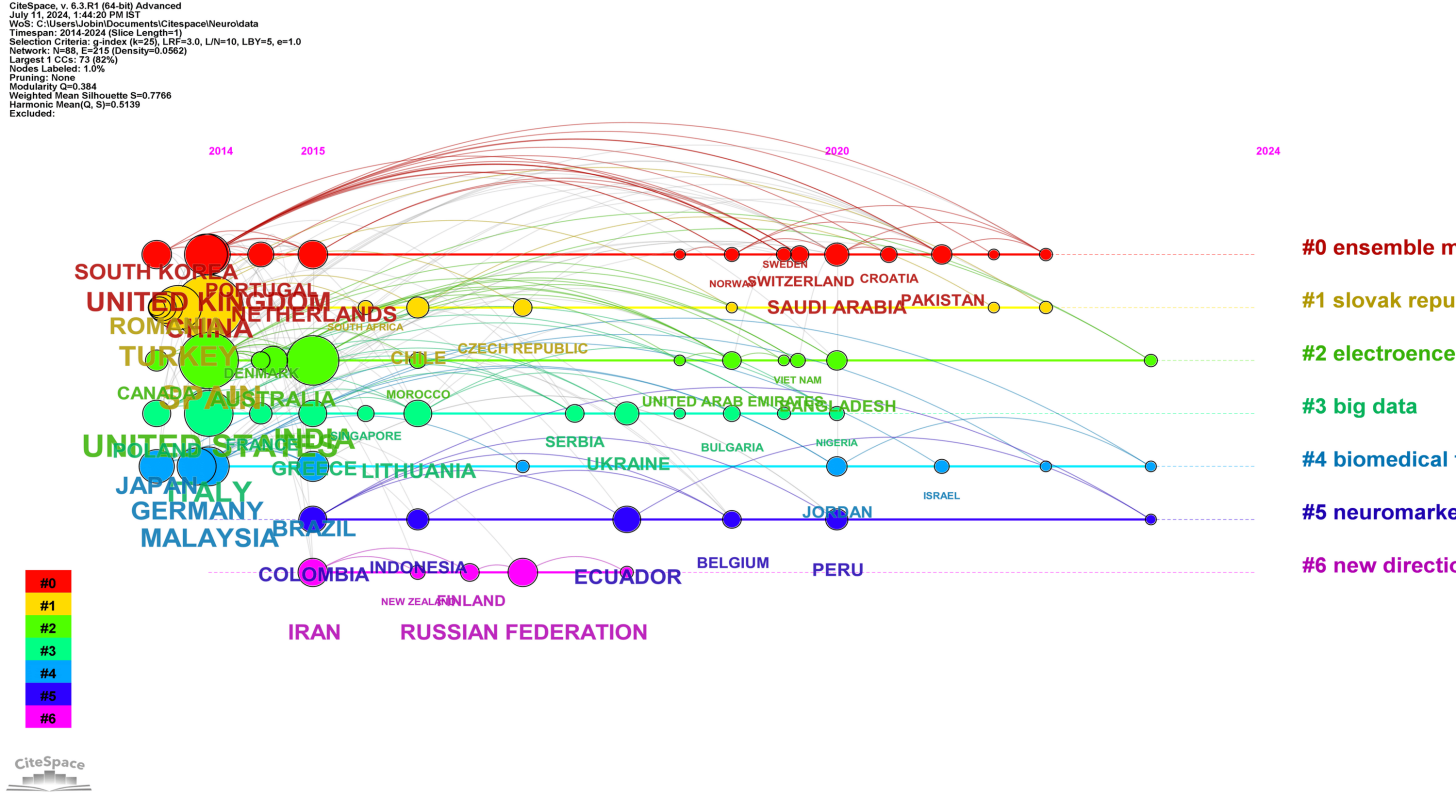
Timeline network visualization of countries' collaborations

The network visualization of countries' collaborations depicted in Figure 4 identifies seven significant clusters, each reflecting distinct collaboration themes and the most active countries within each cluster. Cluster #0, the largest with 15 members, is labeled "ensemble model" and focuses on using EEG signals for systematic investigation. The major cited article in this cluster is by Awan (2022), and the most cited countries are China (47 citations), the United Kingdom (46 citations), and the Netherlands (21 citations). Cluster #1, comprising 13 members, is labeled "Slovak Republic" and centers on user experience and promoting tourist destinations. The most salient citing article comes from Bočková (2021), with Spain being the most cited member (122 citations), followed by Turkey (53 citations) and Romania (27 citations). Cluster #2 regarding a 13-member cluster focused on "electroencephalography signal" and online education. The major citing article comes from Michael (2019), with the most cited countries being the United States (92), followed by India (67) and Australia (20). Cluster #3: "big data" - 12 to develop the neuromarketing concept and going through different mobile applications; major citing article, Ahmed, 2022, with leading countries being Italy with 60 citations, Lithuania 18, and Poland 17. Cluster #4: "biomedical technology" - 9 to research global trends. The most cited article comes from Goto (2019); the most referred-to countries are Malaysia with 40 citations, Germany with 38 citations, and Japan with 31 citations. The sixth cluster, which is six-member in size, is named "neuromarketing y." It comprises works primarily dealing with behavioral economics and the models of attention, emotion, and recall analysis. The largest citing work is from Verdesoto (2023); the central countries cited are Ecuador (17 Citations), Colombia (16 Citations), and Peru (11 Citations). The smallest of the clusters is Cluster #6, which includes five members and is regarding "new direction" and packaging elements. The highest number of major citing articles is the work of Hassani (2023), whereas the most cited countries are the Russian Federation with 20 citations, Iran with 20, and Finland with 7. These clusters underline the variety of international collaborations in neuromarketing research, revealing specific thematic focuses by the cluster as well as country participants.

Table 7 summarizes the clusters identified in the network visualization of countries' collaborations in neuromarketing research. This table provides insights into the dominant collaboration themes and highlights the leading countries in each thematic area within neuromarketing research.

**Table 7:** Countries collaborations

ClusterID	No. of members	Silhouette	Label	Most cited country
0	15	0.731	Ensemble model	China - 47, United Kingdom - 46, Netherlands - 21
1	13	0.686	Slovak republic	Spain - 122, Turkey - 53, Romania - 27
2	13	0.753	Electroencephalography signal	United States - 92, India - 67, Australia - 20
3	12	0.844	Big data	Italy - 60, Lithuania - 18, Poland - 17
4	9	0.838	Biomedical technology	Malaysia - 40, Germany - 38, Japan - 31
5	6	0.812	Neuromarketing	Ecuador - 17, Colombia - 16, Peru - 11
6	5	0.896	New direction	Russian Federation - 20, Iran – 20, Finland - 7

Trend Topics

**Figure 5:**
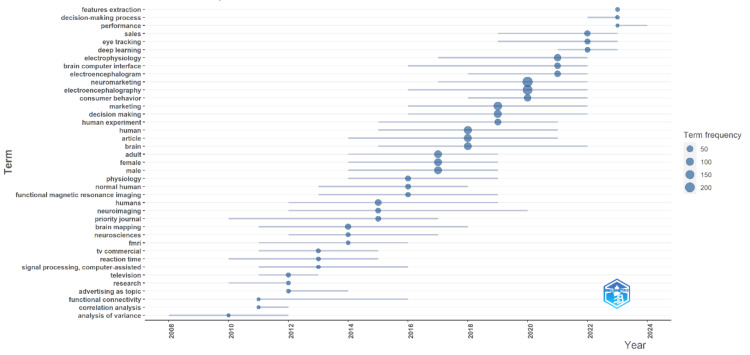
Evolution and frequency of key terms

The trend topic visualization in Figure 5 illustrates the evolution and frequency of key terms in neuromarketing research over time, with circle sizes representing term frequency. In the early years (2008-2012), foundational research and methodological terms such as "functional connectivity," "correlation analysis," and "signal processing" were prominent alongside early applications like "television" and "TV commercials." From 2013 to 2016, the focus shifted towards more specific and advanced topics like "electroencephalography," which began gaining attention around 2016, highlighting the growing interest in neuroimaging techniques and their application in understanding consumer behavior. In contrast, the focus on "electrophysiology" emerged slightly later, around 2017, reflecting a subsequent but significant interest in electrophysiological methods for neuromarketing research. In recent years (2017-2024), there has been a significant increase in the frequency and variety of terms, with advanced technologies and analytical methods like "deep learning," "eye tracking," "brain-computer interface," and "features extraction" becoming prominent. Practical applications and high-impact research terms such as "sales," "performance," and "human experiment" also emerged, indicating the field's maturity. The frequent appearance of "neuromarketing" itself signifies its solid establishment, while other terms like "brain," "human," and "physiology" reflect a broadened research scope encompassing human physiology and brain function in marketing contexts. This dynamic trend underscores the field's evolution towards leveraging sophisticated tools to gain deeper insights into consumer behavior and decision-making processes.

Factorial analysis

**Figure 6:**
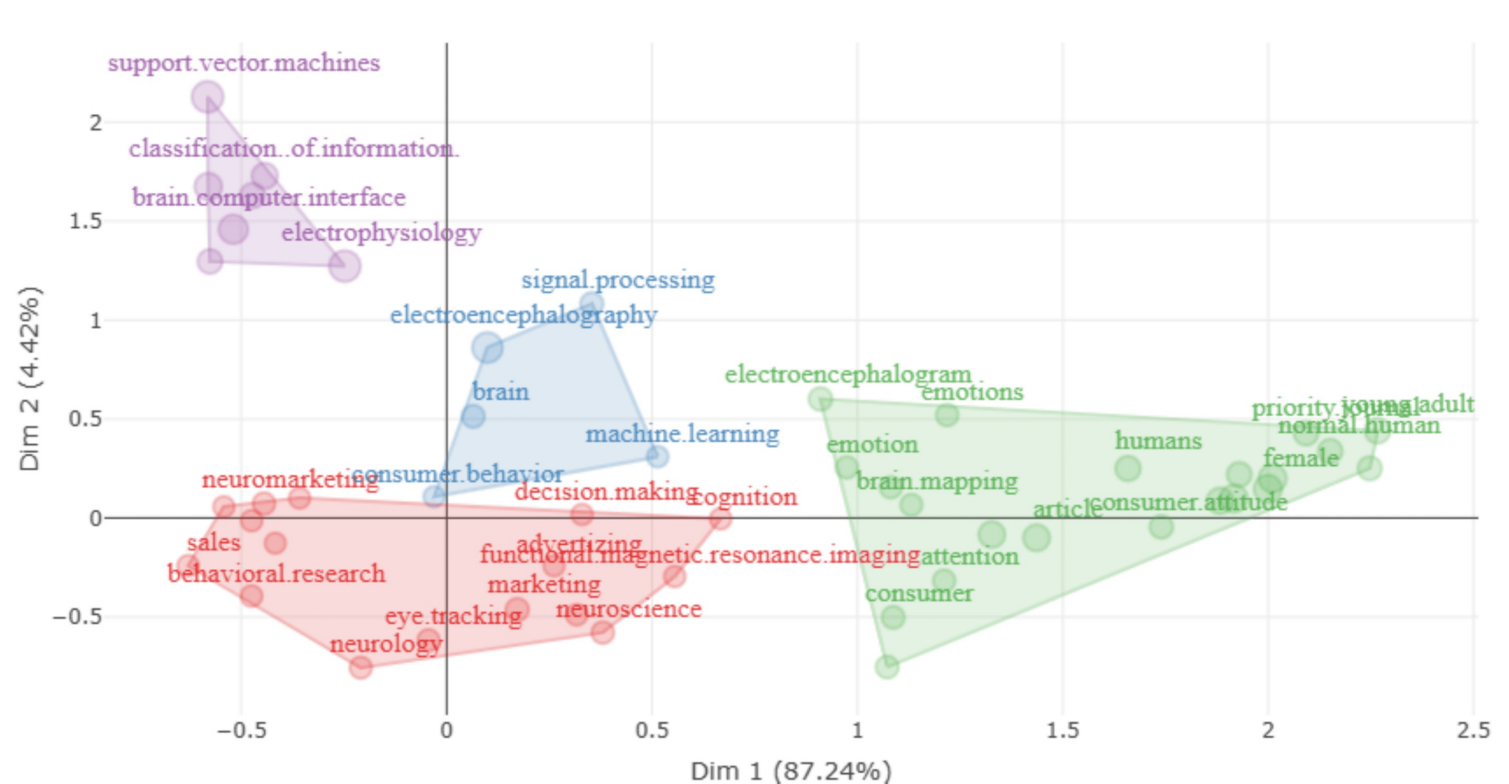
Clustering of research themes

The factorial analysis visualization in Figure 6 shows the clustering of research topics across two dimensions, Dim 1 (87.24%) and Dim 2 (4.42%), indicating how these themes are interrelated based on their co-occurrence in the literature. The analysis reveals four distinct clusters, each representing a unique thematic focus within neuromarketing research. Cluster 1 (purple) encompasses terms like "support vector machines," "classification of information," "brain-computer interface," and "electrophysiology." It focuses on advanced computational techniques and neurotechnology, highlighting the integration of machine learning methods with neurophysiological data to enhance neuromarketing research. Cluster 2 (blue) includes terms such as "signal processing," "electroencephalography," "brain," and "machine learning." This cluster highlights the intersection of signal processing techniques with EEG data analysis, supported by machine learning methods. The focus is on understanding brain activity and its implications for neuromarketing, emphasizing a technical and methodological approach within the field. Cluster 3 (red) covers terms such as "neuromarketing," "consumer behavior," "decision making," "eye tracking," and "functional magnetic resonance imaging (fMRI)." This cluster centers on core neuromarketing topics, with a particular focus on understanding consumer behavior and decision-making processes through the use of neuroimaging techniques like fMRI and eye-tracking technologies. The terms in this cluster highlight the application of neuroscience to marketing strategies, particularly in examining how consumers respond to various marketing stimuli. Cluster 4 (green) features terms like "electroencephalogram," "emotions," and "priority journal," emphasizing the study of emotional responses as measured by EEG. This cluster reflects a research focus on the emotional aspects of consumer behavior, suggesting that understanding emotions is critical for interpreting consumer responses in neuromarketing. The distribution along Dim 1 suggests that this dimension captures the primary methodological differences (e.g., machine learning vs. neuroimaging), while Dim 2 provides additional nuances, distinguishing specific applications or research questions. This analysis underscores the interdisciplinary nature of neuromarketing, integrating diverse methodologies to gain deeper insights into consumer behavior. The clear separation of clusters highlights distinct research trajectories and complementary approaches within the field.

Keywords with the strongest citation bursts

**Figure 7:**
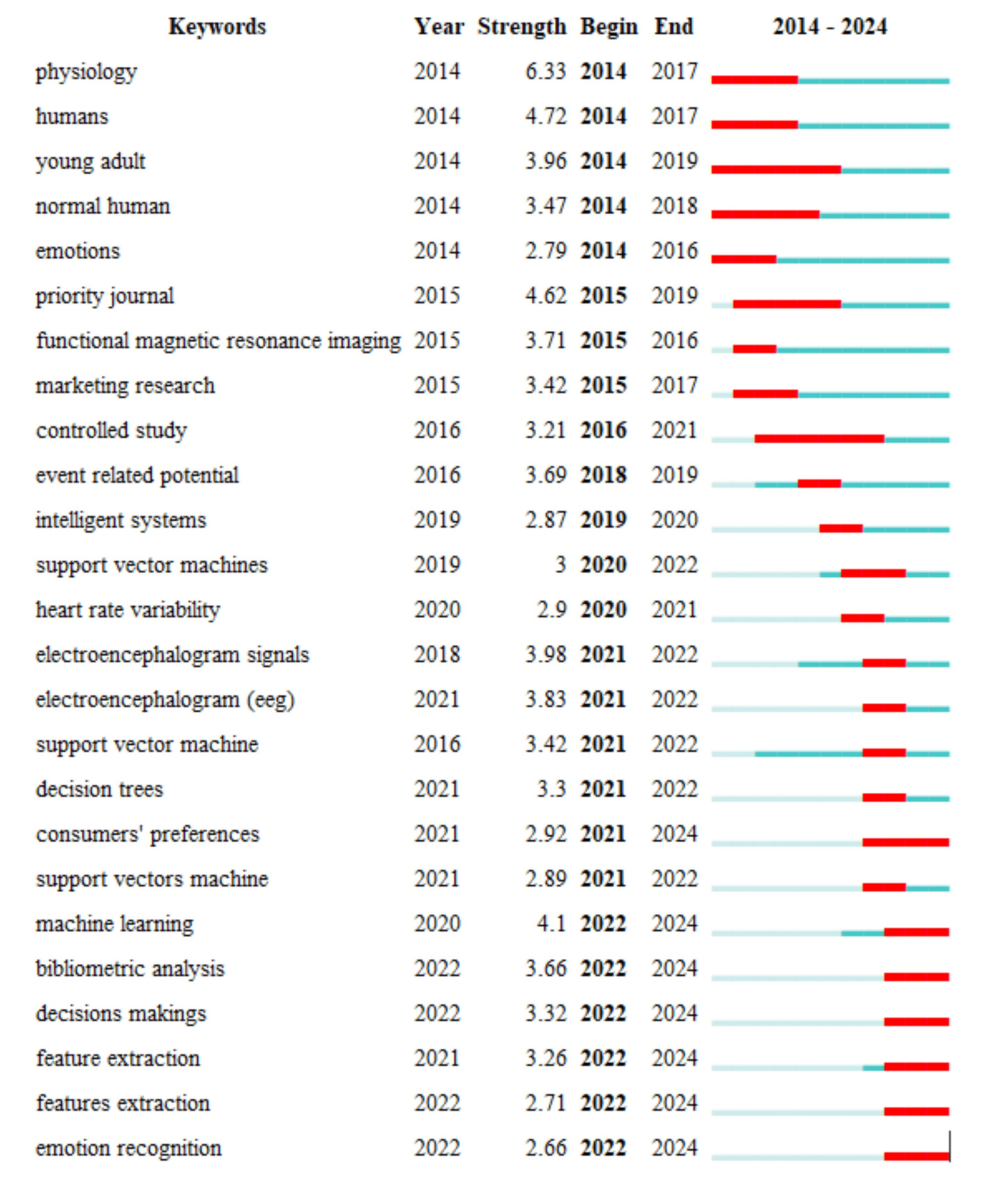
Top 25 keywords with the strongest citation bursts

The visualization in Figure 7 presents the top 25 keywords with the strongest citation bursts, indicating periods of intense research activity and interest in these areas. The keywords are listed with their respective strengths, beginning and ending years, and the duration of their bursts is highlighted. Early keywords such as "physiology," "humans," "young adults," "normal humans," and "emotions" show strong citation bursts starting in 2014, reflecting foundational research interest in human physiology and emotional responses during this period. These bursts typically ended by 2017 or 2018, indicating that these topics were highly influential in the early stages of neuromarketing research. Mid-term keywords like "priority journal," "functional magnetic resonance imaging," "marketing research," and "controlled study" show citation bursts beginning around 2015 and 2016, highlighting a shift towards more specialized and methodological research in neuromarketing. The interest in these topics continued until around 2017 to 2019. Keywords such as "event-related potential" and "intelligent systems" started to gain attention in 2016 and 2019, respectively, indicating an evolving focus on neural response measurement techniques and the application of intelligent systems. More recent keywords include "support vector machines," "heart rate variability," "electroencephalogram signals," "electroencephalogram (EEG)," "decision trees," "consumers' preferences," and "machine learning." These keywords indicate a trend towards integrating advanced computational techniques and machine learning methods in neuromarketing research, starting around 2020 and continuing to the present. The strong bursts of keywords like "support vector machine" and "machine learning" beginning in 2021 highlight the increasing importance of these methods in analyzing consumer data and improving neuromarketing strategies. The latest keywords, such as "bibliometric analysis," "decision making," "feature extraction," and "emotion recognition," show bursts starting in 2022, indicating the most current research trends. The ongoing bursts for these keywords suggest a continuing and growing interest in these areas, reflecting the latest advancements and applications in neuromarketing research. Overall, the timeline and strength of these citation bursts illustrate the evolving focus in neuromarketing research, from foundational physiological studies to the integration of advanced computational techniques and the latest research trends. This analysis highlights the dynamic nature of the field and the shifting priorities in neuromarketing research over the past decade.

Research gaps and practical implications

The bibliometric analysis reveals some research gaps in the field of neuromarketing. Fundamental issues, such as "physiology," "humans," and "emotions," were at the center of researchers' attention at the beginning of the studied period (2014-2017) and decreased at the end of the period, suggesting the need to reconsider these issues with the help of modern research methods to observe more closely what is not visible. For example, "functional magnetic resonance imaging" and "controlled study" under mid-term topics (years 2015- onwards) already indicate a change of window towards specialized and even methodological studies; however, how these are being used and combined in heterogeneous marketing scenarios must be investigated further. Examples of advances in computational methods in recent keywords (last phase from 2020 to 2024) include "support vector machines," "heart rate variability," and "machine learning." However, beyond this, the inquiry is lacking in terms of how these advanced techniques can be applied to marketing's real-world context, particularly considering how advanced methods can be effectively used to drive marketing tactics, strategies, and consumer engagement. Equally important for neuromarketing toward the latest trends up to 2022-2024 is the continuation in area bibliometric analysis of decision-making and emotion recognition.

These new focuses of neuromarketing research, represented by the trend topics and keywords under the most robust citation bursts, entail a series of practical implications for the industry. The recent advanced technologies in "deep learning," "eye tracking," and "brain-computer interface" accord marketers more powerful means to understand consumer behavior and decision-making more profoundly. These findings can apply to companies in building more efficient marketing strategies regarding product placements and increasing consumer engagement. The growth of practical applications and high-impact research terms like "sales," "performance," and "human experiment" indicates that the field has started to mature and has a direct impact on the real world. Marketers should adopt these research findings to design data-driven strategies to improve marketing efficiency and consumer satisfaction. Altogether, this illustrates the last point: the more ever-changing the technology and ways of its marketing application are in their nature and fierceness, the more substantial and more 'bursty' citations within topics like "machine learning" and "emotion recognition" are. Most probably, by addressing the research gaps identified in this survey and leveraging practical implications brought forward by recent findings, neuromarketing will continue to offer valuable insights to marketers dealing with an ever-changing consumer landscape.

Strengths and limitations

This study offers a comprehensive bibliometric analysis of neuromarketing research utilizing the Scopus database, which is renowned for its extensive collection of scientific literature. The combination of Biblioshiny and Citespace tools provides a robust visualization of research trends, key contributors, and collaborative networks, making the findings valuable for understanding the evolution of the field. The systematic approach to data refinement ensures a focus on original research, while the identification of emerging topics through trend analysis and citation bursts sheds light on the dynamic nature of neuromarketing.

However, the study has certain limitations. The exclusive use of the Scopus database may have led to the omission of relevant research from other sources, potentially limiting the comprehensiveness of the analysis. As a bibliometric analysis, this study is inherently more quantitative, focusing on citation counts and publication metrics, which may not fully capture the qualitative aspects of research impact, such as the influence of ideas or the development of new theories.

## Conclusions

This bibliometric insight into neuromarketing highlights the dynamic evolution in this field, driven mainly by integrating modern neuroscience applications and cognitive science techniques. The data were analyzed from Scopus using Biblioshiny and Citespace, and they were analyzed using critical trends, influential authors, pivotal documents, and global research collaborations. The study highlights that the field has reached its point of maturation, on the one hand, because of the broad application of sophisticated methodologies and, on the other hand, because of the rapid growth of a body of high-impact studies. However, there are still some striking gaps in the literature relating to applying sophisticated computational tools in very varied marketing contexts. In this respect, an expanded effort in applying machine learning and deep learning approaches in real neuromarketing situations may further enhance the practical use of these advanced tools. Second, there is a need to enhance interdisciplinary collaborations - all current insights from neuroscience, psychology, and marketing should be integrated together to define a unifying model of consumer behavior. Finally, longitudinal studies need to be conducted for an understanding of the long-term impacts of neuromarketing strategies on consumer behavior and the unraveling of the implications of such approaches for sustainability and ethics in business. By following these recommendations, the neuromarketing field will continue growing, providing increasingly solid and practical insights into consumer behavior to substantiate better the decision process.
